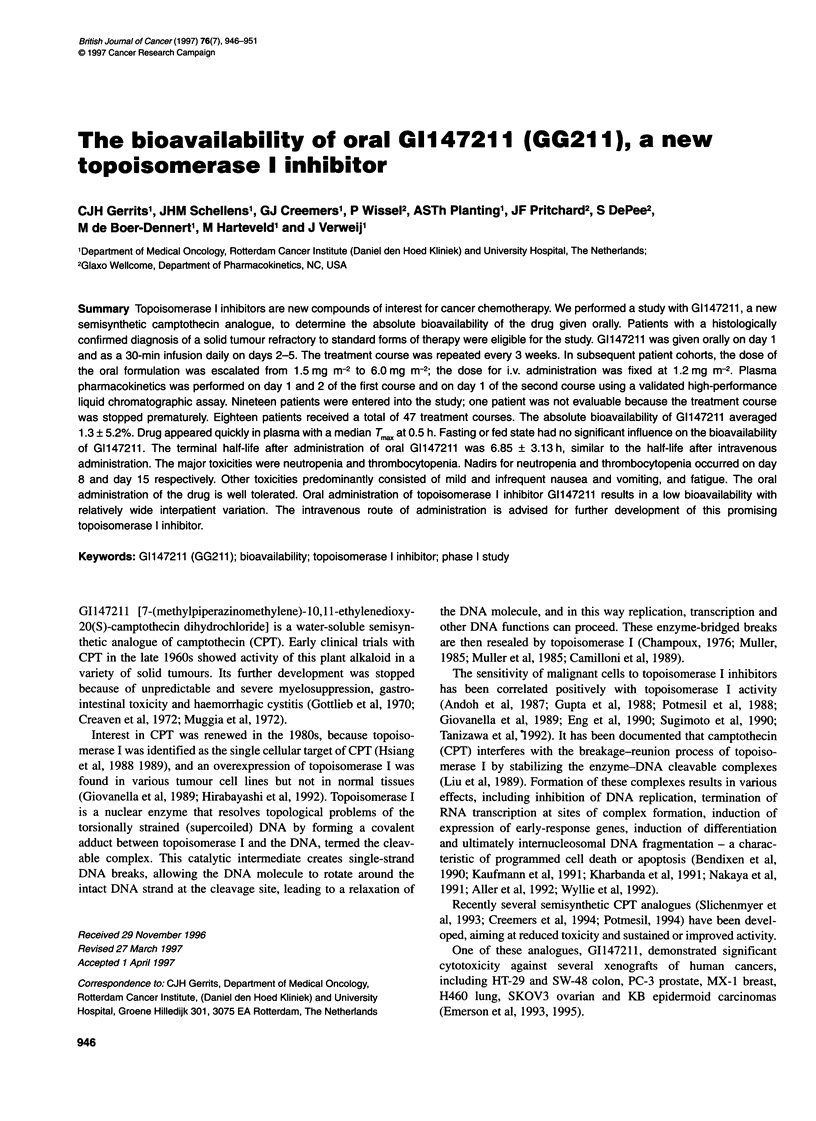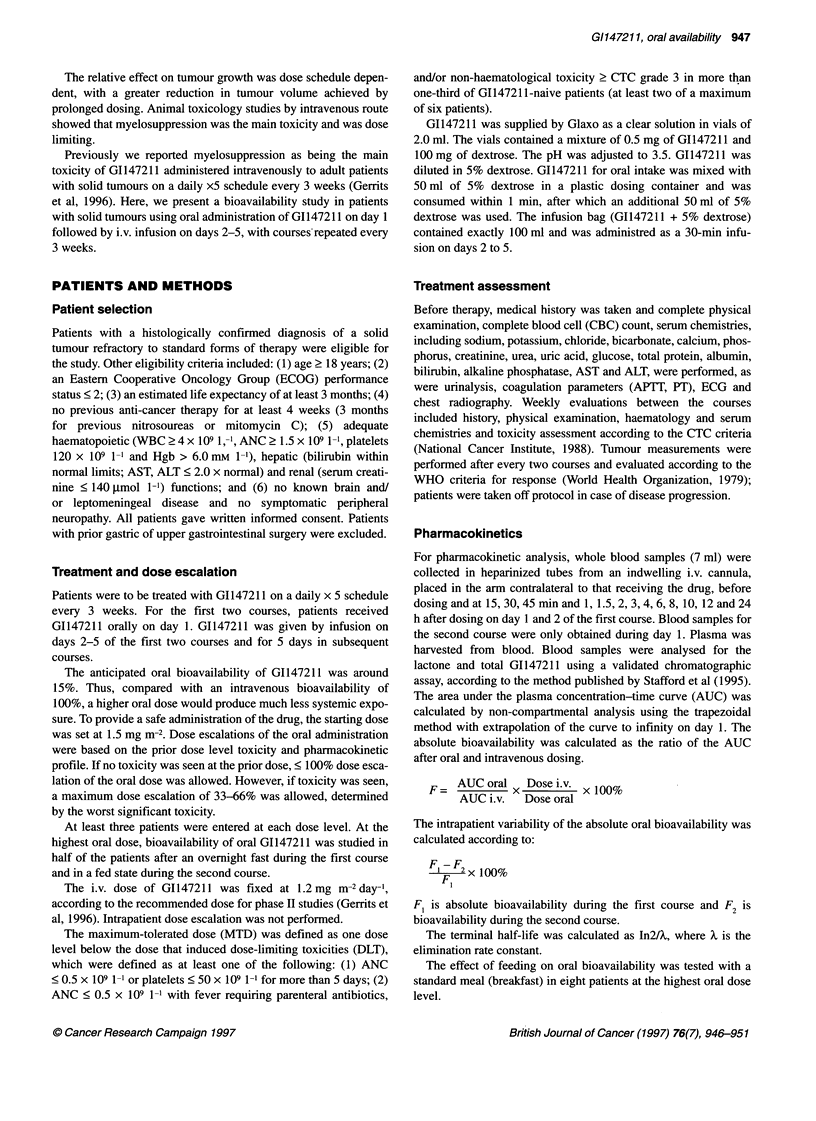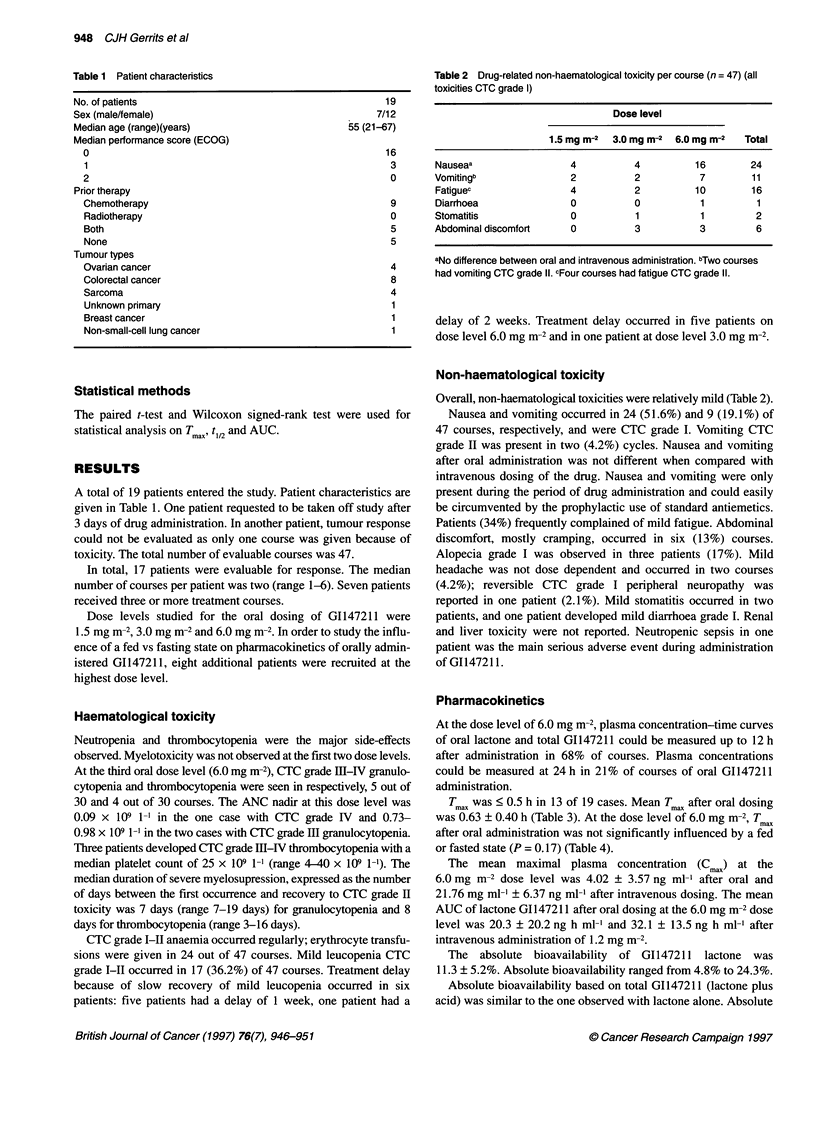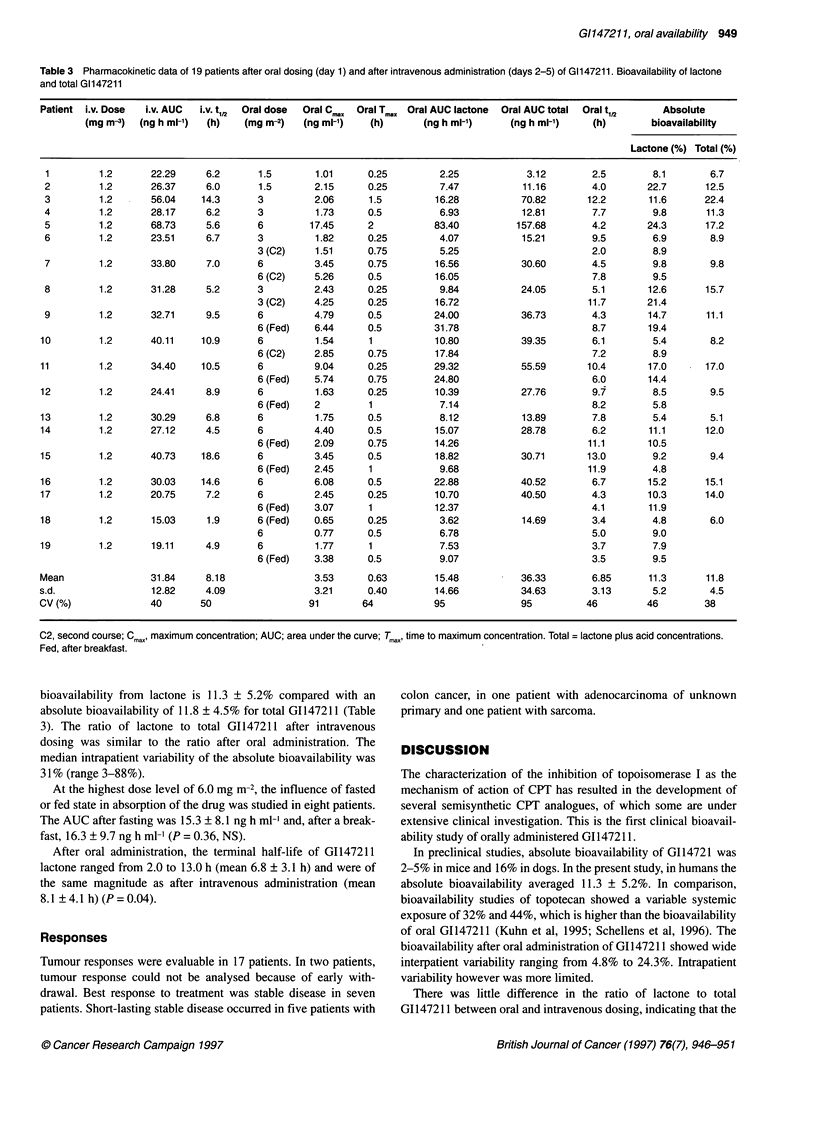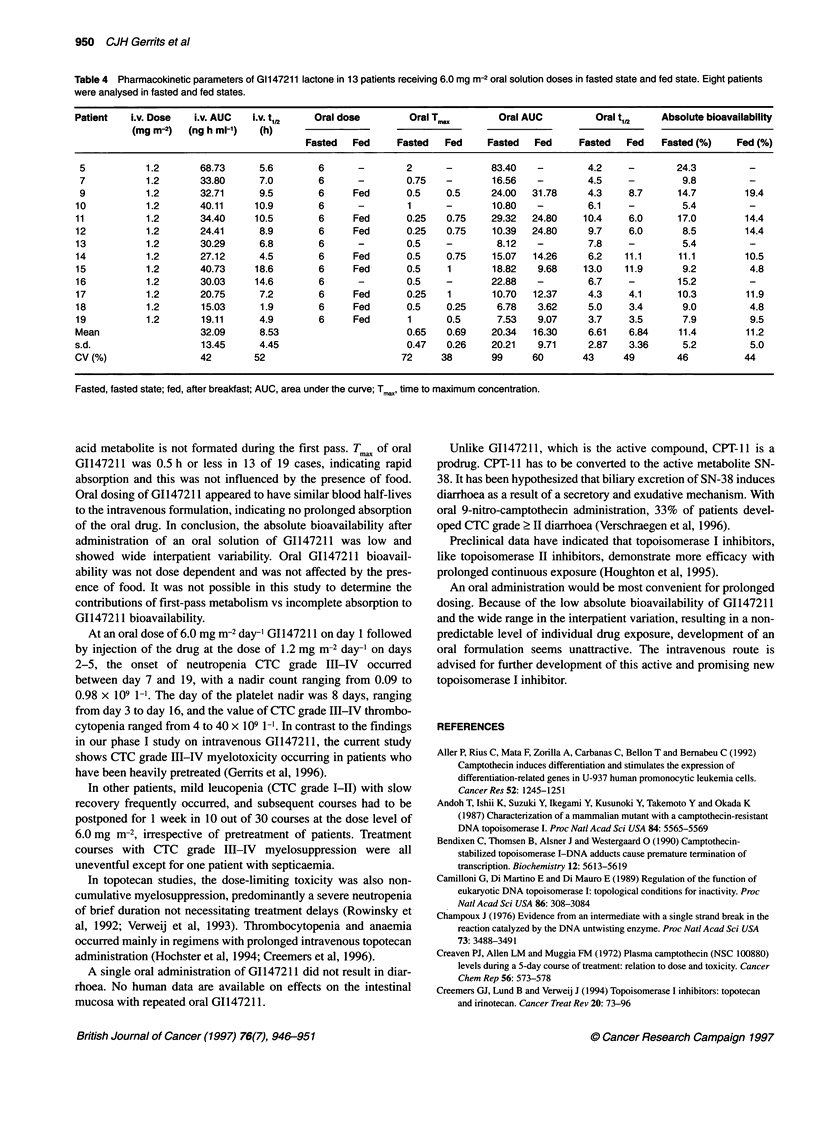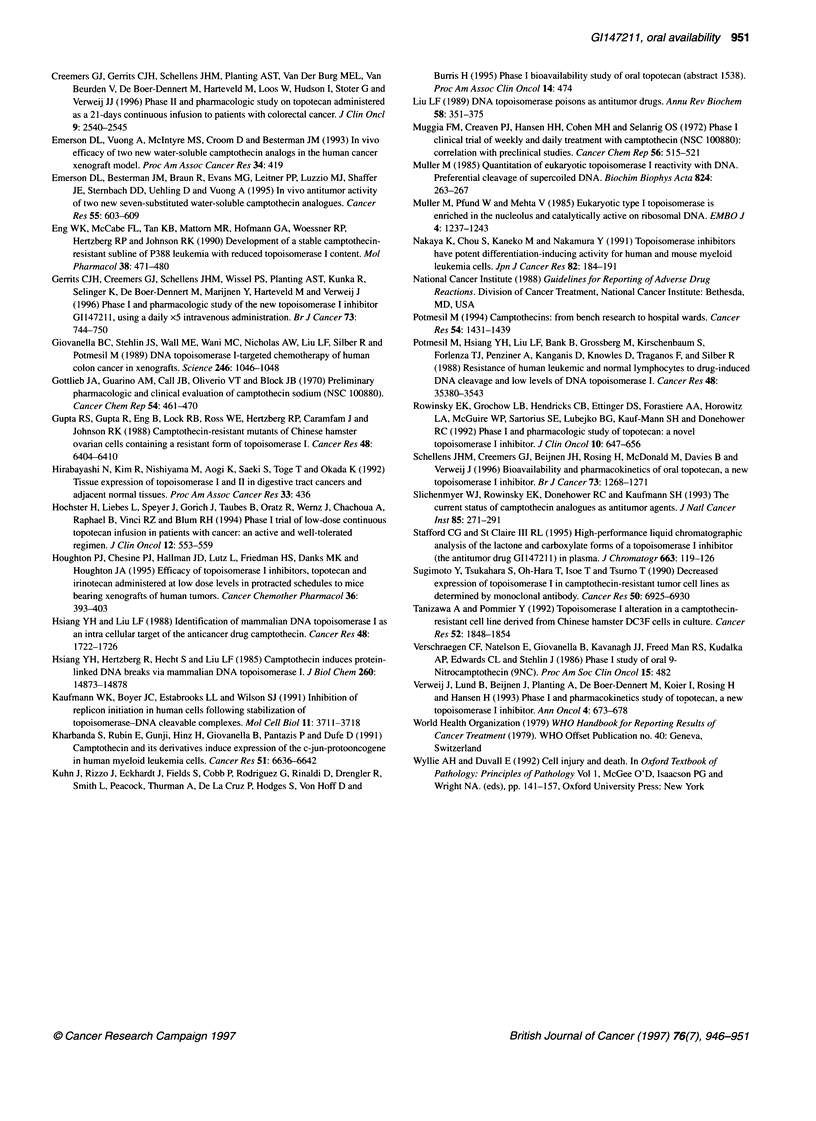# The bioavailability of oral GI147211 (GG211), a new topoisomerase I inhibitor.

**DOI:** 10.1038/bjc.1997.490

**Published:** 1997

**Authors:** C. J. Gerrits, J. H. Schellens, G. J. Creemers, P. Wissel, A. S. Planting, J. F. Pritchard, S. DePee, M. de Boer-Dennert, M. Harteveld, J. Verweij

**Affiliations:** Department of Medical Oncology, Rotterdam Cancer Institute (Daniel den Hoed Kliniek) and University Hospital, The Netherlands.

## Abstract

Topoisomerase I inhibitors are new compounds of interest for cancer chemotherapy. We performed a study with GI147211, a new semisynthetic camptothecin analogue, to determine the absolute bioavailability of the drug given orally. Patients with a histologically confirmed diagnosis of a solid tumour refractory to standard forms of therapy were eligible for the study. GI147211 was given orally on day 1 and as a 30-min infusion daily on days 2-5. The treatment course was repeated every 3 weeks. In subsequent patient cohorts, the dose of the oral formulation was escalated from 1.5 mg m(-2) to 6.0 mg m(-2); the dose for i.v. administration was fixed at 1.2 mg m(-2). Plasma pharmacokinetics was performed on day 1 and 2 of the first course and on day 1 of the second course using a validated high-performance liquid chromatographic assay. Nineteen patients were entered into the study; one patient was not evaluable because the treatment course was stopped prematurely. Eighteen patients received a total of 47 treatment courses. The absolute bioavailability of GI147211 averaged 1.3 +/- 5.2%. Drug appeared quickly in plasma with a median Tmax at 0.5 h. Fasting or fed state had no significant influence on the bioavailability of GI147211. The terminal half-life after administration of oral GI147211 was 6.85 +/- 3.13 h, similar to the half-life after intravenous administration. The major toxicities were neutropenia and thrombocytopenia. Nadirs for neutropenia and thrombocytopenia occurred on day 8 and day 15 respectively. Other toxicities predominantly consisted of mild and infrequent nausea and vomiting, and fatigue. The oral administration of the drug is well tolerated. Oral administration of topoisomerase I inhibitor GI147211 results in a low bioavailability with relatively wide interpatient variation. The intravenous route of administration is advised for further development of this promising topoisomerase I inhibitor.


British Journal of Cancer (1997) 76(7), 946–951
© 1997 Cancer Research Campaign

### The bioavailability of oral GI147211 (GG211), a new topoisomerase I inhibitor

**CJH Gerrits[1], JHM Schellens[1], GJ Creemers[1], P Wissel[2], ASTh Planting[1], JF Pritchard[2], S DePee[2], M de Boer-Dennert[1], M Harteveld[1] and J Verweij[1]**

[1]Department of Medical Oncology, Rotterdam Cancer Institute (Daniel den Hoed Kliniek) and University Hospital, The Netherlands;
[2]Glaxo Wellcome, Department of Pharmacokinetics, NC, USA

**Summary** Topoisomerase I inhibitors are new compounds of interest for cancer chemotherapy. We performed a study with GI147211, a new semisynthetic camptothecin analogue, to determine the absolute bioavailability of the drug given orally. Patients with a histologically confirmed diagnosis of a solid tumour refractory to standard forms of therapy were eligible for the study. GI147211 was given orally on day 1 and as a 30-min infusion daily on days 2–5. The treatment course was repeated every 3 weeks. In subsequent patient cohorts, the dose of the oral formulation was escalated from 1.5 mg $m^{-2}$ to 6.0 mg $m^{-2}$; the dose for i.v. administration was fixed at 1.2 mg $m^{-2}$. Plasma pharmacokinetics was performed on day 1 and 2 of the first course and on day 1 of the second course using a validated high-performance liquid chromatographic assay. Nineteen patients were entered into the study; one patient was not evaluable because the treatment course was stopped prematurely. Eighteen patients received a total of 47 treatment courses. The absolute bioavailability of GI147211 averaged 1.3 ± 5.2%. Drug appeared quickly in plasma with a median $T_{max}$ at 0.5 h. Fasting or fed state had no significant influence on the bioavailability of GI147211. The terminal half-life after administration of oral GI147211 was 6.85 ± 3.13 h, similar to the half-life after intravenous administration. The major toxicities were neutropenia and thrombocytopenia. Nadirs for neutropenia and thrombocytopenia occurred on day 8 and day 15 respectively. Other toxicities predominantly consisted of mild and infrequent nausea and vomiting, and fatigue. The oral administration of the drug is well tolerated. Oral administration of topoisomerase I inhibitor GI147211 results in a low bioavailability with relatively wide interpatient variation. The intravenous route of administration is advised for further development of this promising topoisomerase I inhibitor.

**Keywords:** GI147211 (GG211); bioavailability; topoisomerase I inhibitor; phase I study

GI147211 [7-(methylpiperazinomethylene)-10,11-ethylenedioxy-20(S)-camptothecin dihydrochloride] is a water-soluble semisynthetic analogue of camptothecin (CPT). Early clinical trials with CPT in the late 1960s showed activity of this plant alkaloid in a variety of solid tumours. Its further development was stopped because of unpredictable and severe myelosuppression, gastrointestinal toxicity and haemorrhagic cystitis (Gottlieb et al, 1970; Creaven et al, 1972; Muggia et al, 1972).

Interest in CPT was renewed in the 1980s, because topoisomerase I was identified as the single cellular target of CPT (Hsiang et al, 1988 1989), and an overexpression of topoisomerase I was found in various tumour cell lines but not in normal tissues (Giovanella et al, 1989; Hirabayashi et al, 1992). Topoisomerase I is a nuclear enzyme that resolves topological problems of the torsionally strained (supercoiled) DNA by forming a covalent adduct between topoisomerase I and the DNA, termed the cleavable complex. This catalytic intermediate creates single-strand DNA breaks, allowing the DNA molecule to rotate around the intact DNA strand at the cleavage site, leading to a relaxation of the DNA molecule, and in this way replication, transcription and other DNA functions can proceed. These enzyme-bridged breaks are then resealed by topoisomerase I (Champoux, 1976; Muller, 1985; Muller et al, 1985; Camilloni et al, 1989).

The sensitivity of malignant cells to topoisomerase I inhibitors has been correlated positively with topoisomerase I activity (Andoh et al, 1987; Gupta et al, 1988; Potmesil et al, 1988; Giovanella et al, 1989; Eng et al, 1990; Sugimoto et al, 1990; Tanizawa et al, 1992). It has been documented that camptothecin (CPT) interferes with the breakage–reunion process of topoisomerase I by stabilizing the enzyme–DNA cleavable complexes (Liu et al, 1989). Formation of these complexes results in various effects, including inhibition of DNA replication, termination of RNA transcription at sites of complex formation, induction of expression of early-response genes, induction of differentiation and ultimately internucleosomal DNA fragmentation – a characteristic of programmed cell death or apoptosis (Bendixen et al, 1990; Kaufmann et al, 1991; Kharbanda et al, 1991; Nakaya et al, 1991; Aller et al, 1992; Wyllie et al, 1992).

Recently several semisynthetic CPT analogues (Slichenmyer et al, 1993; Creemers et al, 1994; Potmesil, 1994) have been developed, aiming at reduced toxicity and sustained or improved activity.

One of these analogues, GI147211, demonstrated significant cytotoxicity against several xenografts of human cancers, including HT-29 and SW-48 colon, PC-3 prostate, MX-1 breast, H460 lung, SKOV3 ovarian and KB epidermoid carcinomas (Emerson et al, 1993, 1995).

*Received 29 November 1996*
*Revised 27 March 1997*
*Accepted 1 April 1997*

*Correspondence to:* CJH Gerrits, Department of Medical Oncology, Rotterdam Cancer Institute, (Daniel den Hoed Kliniek) and University Hospital, Groene Hilledijk 301, 3075 EA Rotterdam, The Netherlands

The relative effect on tumour growth was dose schedule dependent, with a greater reduction in tumour volume achieved by prolonged dosing. Animal toxicology studies by intravenous route showed that myelosuppression was the main toxicity and was dose limiting.

Previously we reported myelosuppression as being the main toxicity of GI147211 administered intravenously to adult patients with solid tumours on a daily ×5 schedule every 3 weeks (Gerrits et al, 1996). Here, we present a bioavailability study in patients with solid tumours using oral administration of GI147211 on day 1 followed by i.v. infusion on days 2–5, with courses repeated every 3 weeks.

### PATIENTS AND METHODS

### Patient selection

Patients with a histologically confirmed diagnosis of a solid tumour refractory to standard forms of therapy were eligible for the study. Other eligibility criteria included: (1) age ≥ 18 years; (2) an Eastern Cooperative Oncology Group (ECOG) performance status ≤ 2; (3) an estimated life expectancy of at least 3 months; (4) no previous anti-cancer therapy for at least 4 weeks (3 months for previous nitrosoureas or mitomycin C); (5) adequate haematopoietic (WBC ≥ $4 \times 10^9$ $1,^{-1}$, ANC ≥ $1.5 \times 10^9$ $1^{-1}$, platelets $120 \times 10^9$ $1^{-1}$ and Hgb > 6.0 mM $1^{-1}$), hepatic (bilirubin within normal limits; AST, ALT ≤ 2.0 × normal) and renal (serum creatinine ≤ 140 μmol $1^{-1}$) functions; and (6) no known brain and/or leptomeningeal disease and no symptomatic peripheral neuropathy. All patients gave written informed consent. Patients with prior gastric of upper gastrointestinal surgery were excluded.

### Treatment and dose escalation

Patients were to be treated with GI147211 on a daily × 5 schedule every 3 weeks. For the first two courses, patients received GI147211 orally on day 1. GI147211 was given by infusion on days 2–5 of the first two courses and for 5 days in subsequent courses.

The anticipated oral bioavailability of GI147211 was around 15%. Thus, compared with an intravenous bioavailability of 100%, a higher oral dose would produce much less systemic exposure. To provide a safe administration of the drug, the starting dose was set at 1.5 mg $m^{-2}$. Dose escalations of the oral administration were based on the prior dose level toxicity and pharmacokinetic profile. If no toxicity was seen at the prior dose, ≤ 100% dose escalation of the oral dose was allowed. However, if toxicity was seen, a maximum dose escalation of 33–66% was allowed, determined by the worst significant toxicity.

At least three patients were entered at each dose level. At the highest oral dose, bioavailability of oral GI147211 was studied in half of the patients after an overnight fast during the first course and in a fed state during the second course.

The i.v. dose of GI147211 was fixed at 1.2 mg $m^{-2}$ $day^{-1}$, according to the recommended dose for phase II studies (Gerrits et al, 1996). Intrapatient dose escalation was not performed.

The maximum-tolerated dose (MTD) was defined as one dose level below the dose that induced dose-limiting toxicities (DLT), which were defined as at least one of the following: (1) ANC ≤ $0.5 \times 10^9$ $1^{-1}$ or platelets ≤ $50 \times 10^9$ $1^{-1}$ for more than 5 days; (2) ANC ≤ $0.5 \times 10^9$ $1^{-1}$ with fever requiring parenteral antibiotics, and/or non-haematological toxicity ≥ CTC grade 3 in more than one-third of GI147211-naive patients (at least two of a maximum of six patients).

GI147211 was supplied by Glaxo as a clear solution in vials of 2.0 ml. The vials contained a mixture of 0.5 mg of GI147211 and 100 mg of dextrose. The pH was adjusted to 3.5. GI147211 was diluted in 5% dextrose. GI147211 for oral intake was mixed with 50 ml of 5% dextrose in a plastic dosing container and was consumed within 1 min, after which an additional 50 ml of 5% dextrose was used. The infusion bag (GI147211 + 5% dextrose) contained exactly 100 ml and was administred as a 30-min infusion on days 2 to 5.

### Treatment assessment

Before therapy, medical history was taken and complete physical examination, complete blood cell (CBC) count, serum chemistries, including sodium, potassium, chloride, bicarbonate, calcium, phosphorus, creatinine, urea, uric acid, glucose, total protein, albumin, bilirubin, alkaline phosphatase, AST and ALT, were performed, as were urinalysis, coagulation parameters (APTT, PT), ECG and chest radiography. Weekly evaluations between the courses included history, physical examination, haematology and serum chemistries and toxicity assessment according to the CTC criteria (National Cancer Institute, 1988). Tumour measurements were performed after every two courses and evaluated according to the WHO criteria for response (World Health Organization, 1979); patients were taken off protocol in case of disease progression.

### Pharmacokinetics

For pharmacokinetic analysis, whole blood samples (7 ml) were collected in heparinized tubes from an indwelling i.v. cannula, placed in the arm contralateral to that receiving the drug, before dosing and at 15, 30, 45 min and 1, 1.5, 2, 3, 4, 6, 8, 10, 12 and 24 h after dosing on day 1 and 2 of the first course. Blood samples for the second course were only obtained during day 1. Plasma was harvested from blood. Blood samples were analysed for the lactone and total GI147211 using a validated chromatographic assay, according to the method published by Stafford et al (1995). The area under the plasma concentration–time curve (AUC) was calculated by non-compartmental analysis using the trapezoidal method with extrapolation of the curve to infinity on day 1. The absolute bioavailability was calculated as the ratio of the AUC after oral and intravenous dosing.

$$F = \frac{\text{AUC oral}}{\text{AUC i.v.}} \times \frac{\text{Dose i.v.}}{\text{Dose oral}} \times 100\%$$

The intrapatient variability of the absolute oral bioavailability was calculated according to:

$$\frac{F_1 - F_2}{F_1} \times 100\%$$

$F_1$ is absolute bioavailability during the first course and $F_2$ is bioavailability during the second course.

The terminal half-life was calculated as In2/λ, where λ is the elimination rate constant.

The effect of feeding on oral bioavailability was tested with a standard meal (breakfast) in eight patients at the highest oral dose level.

© Cancer Research Campaign 1997

**Table 1** Patient characteristics

| | |
|---|---|
| No. of patients | 19 |
| Sex (male/female) | 7/12 |
| Median age (range)(years) | 55 (21–67) |
| Median performance score (ECOG) | |
| 0 | 16 |
| 1 | 3 |
| 2 | 0 |
| Prior therapy | |
| Chemotherapy | 9 |
| Radiotherapy | 0 |
| Both | 5 |
| None | 5 |
| Tumour types | |
| Ovarian cancer | 4 |
| Colorectal cancer | 8 |
| Sarcoma | 4 |
| Unknown primary | 1 |
| Breast cancer | 1 |
| Non-small-cell lung cancer | 1 |

### Statistical methods

The paired *t*-test and Wilcoxon signed-rank test were used for statistical analysis on $T_{max}$, $t_{1/2}$ and AUC.

### RESULTS

A total of 19 patients entered the study. Patient characteristics are given in Table 1. One patient requested to be taken off study after 3 days of drug administration. In another patient, tumour response could not be evaluated as only one course was given because of toxicity. The total number of evaluable courses was 47.

In total, 17 patients were evaluable for response. The median number of courses per patient was two (range 1–6). Seven patients received three or more treatment courses.

Dose levels studied for the oral dosing of GI147211 were 1.5 mg $m^{-2}$, 3.0 mg $m^{-2}$ and 6.0 mg $m^{-2}$. In order to study the influence of a fed vs fasting state on pharmacokinetics of orally administered GI147211, eight additional patients were recruited at the highest dose level.

### Haematological toxicity

Neutropenia and thrombocytopenia were the major side-effects observed. Myelotoxicity was not observed at the first two dose levels. At the third oral dose level (6.0 mg $m^{-2}$), CTC grade III–IV granulocytopenia and thrombocytopenia were seen in respectively, 5 out of 30 and 4 out of 30 courses. The ANC nadir at this dose level was $0.09 \times 10^9$ $l^{-1}$ in the one case with CTC grade IV and 0.73–$0.98 \times 10^9$ $l^{-1}$ in the two cases with CTC grade III granulocytopenia. Three patients developed CTC grade III–IV thrombocytopenia with a median platelet count of $25 \times 10^9$ $l^{-1}$ (range 4–$40 \times 10^9$ $l^{-1}$). The median duration of severe myelosupression, expressed as the number of days between the first occurrence and recovery to CTC grade II toxicity was 7 days (range 7–19 days) for granulocytopenia and 8 days for thrombocytopenia (range 3–16 days).

CTC grade I–II anaemia occurred regularly; erythrocyte transfusions were given in 24 out of 47 courses. Mild leucopenia CTC grade I–II occurred in 17 (36.2%) of 47 courses. Treatment delay because of slow recovery of mild leucopenia occurred in six patients: five patients had a delay of 1 week, one patient had a delay of 2 weeks. Treatment delay occurred in five patients on dose level 6.0 mg $m^{-2}$ and in one patient at dose level 3.0 mg $m^{-2}$.

**Table 2** Drug-related non-haematological toxicity per course ($n = 47$) (all toxicities CTC grade I)

| | Dose level | | | |
|---|---|---|---|---|
| | 1.5 mg $m^{-2}$ | 3.0 mg $m^{-2}$ | 6.0 mg $m^{-2}$ | Total |
| Nausea[a] | 4 | 4 | 16 | 24 |
| Vomiting[b] | 2 | 2 | 7 | 11 |
| Fatigue[c] | 4 | 2 | 10 | 16 |
| Diarrhoea | 0 | 0 | 1 | 1 |
| Stomatitis | 0 | 1 | 1 | 2 |
| Abdominal discomfort | 0 | 3 | 3 | 6 |

[a]No difference between oral and intravenous administration. [b]Two courses had vomiting CTC grade II. [c]Four courses had fatigue CTC grade II.

### Non-haematological toxicity

Overall, non-haematological toxicities were relatively mild (Table 2).

Nausea and vomiting occurred in 24 (51.6%) and 9 (19.1%) of 47 courses, respectively, and were CTC grade I. Vomiting CTC grade II was present in two (4.2%) cycles. Nausea and vomiting after oral administration was not different when compared with intravenous dosing of the drug. Nausea and vomiting were only present during the period of drug administration and could easily be circumvented by the prophylactic use of standard antiemetics. Patients (34%) frequently complained of mild fatigue. Abdominal discomfort, mostly cramping, occurred in six (13%) courses. Alopecia grade I was observed in three patients (17%). Mild headache was not dose dependent and occurred in two courses (4.2%); reversible CTC grade I peripheral neuropathy was reported in one patient (2.1%). Mild stomatitis occurred in two patients, and one patient developed mild diarrhoea grade I. Renal and liver toxicity were not reported. Neutropenic sepsis in one patient was the main serious adverse event during administration of GI147211.

### Pharmacokinetics

At the dose level of 6.0 mg $m^{-2}$, plasma concentration–time curves of oral lactone and total GI147211 could be measured up to 12 h after administration in 68% of courses. Plasma concentrations could be measured at 24 h in 21% of courses of oral GI147211 administration.

$T_{max}$ was $\leq 0.5$ h in 13 of 19 cases. Mean $T_{max}$ after oral dosing was $0.63 \pm 0.40$ h (Table 3). At the dose level of 6.0 mg $m^{-2}$, $T_{max}$ after oral administration was not significantly influenced by a fed or fasted state ($P = 0.17$) (Table 4).

The mean maximal plasma concentration ($C_{max}$) at the 6.0 mg $m^{-2}$ dose level was $4.02 \pm 3.57$ ng $ml^{-1}$ after oral and 21.76 mg $ml^{-1}$ $\pm$ 6.37 ng $ml^{-1}$ after intravenous dosing. The mean AUC of lactone GI147211 after oral dosing at the 6.0 mg $m^{-2}$ dose level was $20.3 \pm 20.2$ ng h $ml^{-1}$ and $32.1 \pm 13.5$ ng h $ml^{-1}$ after intravenous administration of 1.2 mg $m^{-2}$.

The absolute bioavailability of GI147211 lactone was $11.3 \pm 5.2\%$. Absolute bioavailability ranged from 4.8% to 24.3%.

Absolute bioavailability based on total GI147211 (lactone plus acid) was similar to the one observed with lactone alone. Absolute

© Cancer Research Campaign 1997

**Table 3** Pharmacokinetic data of 19 patients after oral dosing (day 1) and after intravenous administration (days 2–5) of GI147211. Bioavailability of lactone and total GI147211

| Patient | i.v. Dose (mg m$^{-3}$) | i.v. AUC (ng h ml$^{-1}$) | i.v. $t_{1/2}$ (h) | Oral dose (mg m$^{-2}$) | Oral $C_{max}$ (ng ml$^{-1}$) | Oral $T_{max}$ (h) | Oral AUC lactone (ng h ml$^{-1}$) | Oral AUC total (ng h ml$^{-1}$) | Oral $t_{1/2}$ (h) | Absolute bioavailability | |
|---|---|---|---|---|---|---|---|---|---|---|---|
| | | | | | | | | | | Lactone (%) | Total (%) |
| 1 | 1.2 | 22.29 | 6.2 | 1.5 | 1.01 | 0.25 | 2.25 | 3.12 | 2.5 | 8.1 | 6.7 |
| 2 | 1.2 | 26.37 | 6.0 | 1.5 | 2.15 | 0.25 | 7.47 | 11.16 | 4.0 | 22.7 | 12.5 |
| 3 | 1.2 | 56.04 | 14.3 | 3 | 2.06 | 1.5 | 16.28 | 70.82 | 12.2 | 11.6 | 22.4 |
| 4 | 1.2 | 28.17 | 6.2 | 3 | 1.73 | 0.5 | 6.93 | 12.81 | 7.7 | 9.8 | 11.3 |
| 5 | 1.2 | 68.73 | 5.6 | 6 | 17.45 | 2 | 83.40 | 157.68 | 4.2 | 24.3 | 17.2 |
| 6 | 1.2 | 23.51 | 6.7 | 3 | 1.82 | 0.25 | 4.07 | 15.21 | 9.5 | 6.9 | 8.9 |
| | | | | 3 (C2) | 1.51 | 0.75 | 5.25 | | 2.0 | 8.9 | |
| 7 | 1.2 | 33.80 | 7.0 | 6 | 3.45 | 0.75 | 16.56 | 30.60 | 4.5 | 9.8 | 9.8 |
| | | | | 6 (C2) | 5.26 | 0.5 | 16.05 | | 7.8 | 9.5 | |
| 8 | 1.2 | 31.28 | 5.2 | 3 | 2.43 | 0.25 | 9.84 | 24.05 | 5.1 | 12.6 | 15.7 |
| | | | | 3 (C2) | 4.25 | 0.25 | 16.72 | | 11.7 | 21.4 | |
| 9 | 1.2 | 32.71 | 9.5 | 6 | 4.79 | 0.5 | 24.00 | 36.73 | 4.3 | 14.7 | 11.1 |
| | | | | 6 (Fed) | 6.44 | 0.5 | 31.78 | | 8.7 | 19.4 | |
| 10 | 1.2 | 40.11 | 10.9 | 6 | 1.54 | 1 | 10.80 | 39.35 | 6.1 | 5.4 | 8.2 |
| | | | | 6 (C2) | 2.85 | 0.75 | 17.84 | | 7.2 | 8.9 | |
| 11 | 1.2 | 34.40 | 10.5 | 6 | 9.04 | 0.25 | 29.32 | 55.59 | 10.4 | 17.0 | 17.0 |
| | | | | 6 (Fed) | 5.74 | 0.75 | 24.80 | | 6.0 | 14.4 | |
| 12 | 1.2 | 24.41 | 8.9 | 6 | 1.63 | 0.25 | 10.39 | 27.76 | 9.7 | 8.5 | 9.5 |
| | | | | 6 (Fed) | 2 | 1 | 7.14 | | 8.2 | 5.8 | |
| 13 | 1.2 | 30.29 | 6.8 | 6 | 1.75 | 0.5 | 8.12 | 13.89 | 7.8 | 5.4 | 5.1 |
| 14 | 1.2 | 27.12 | 4.5 | 6 | 4.40 | 0.5 | 15.07 | 28.78 | 6.2 | 11.1 | 12.0 |
| | | | | 6 (Fed) | 2.09 | 0.75 | 14.26 | | 11.1 | 10.5 | |
| 15 | 1.2 | 40.73 | 18.6 | 6 | 3.45 | 0.5 | 18.82 | 30.71 | 13.0 | 9.2 | 9.4 |
| | | | | 6 (Fed) | 2.45 | 1 | 9.68 | | 11.9 | 4.8 | |
| 16 | 1.2 | 30.03 | 14.6 | 6 | 6.08 | 0.5 | 22.88 | 40.52 | 6.7 | 15.2 | 15.1 |
| 17 | 1.2 | 20.75 | 7.2 | 6 | 2.45 | 0.25 | 10.70 | 40.50 | 4.3 | 10.3 | 14.0 |
| | | | | 6 (Fed) | 3.07 | 1 | 12.37 | | 4.1 | 11.9 | |
| 18 | 1.2 | 15.03 | 1.9 | 6 (Fed) | 0.65 | 0.25 | 3.62 | 14.69 | 3.4 | 4.8 | 6.0 |
| | | | | 6 | 0.77 | 0.5 | 6.78 | | 5.0 | 9.0 | |
| 19 | 1.2 | 19.11 | 4.9 | 6 | 1.77 | 1 | 7.53 | | 3.7 | 7.9 | |
| | | | | 6 (Fed) | 3.38 | 0.5 | 9.07 | | 3.5 | 9.5 | |
| Mean | | 31.84 | 8.18 | | 3.53 | 0.63 | 15.48 | 36.33 | 6.85 | 11.3 | 11.8 |
| s.d. | | 12.82 | 4.09 | | 3.21 | 0.40 | 14.66 | 34.63 | 3.13 | 5.2 | 4.5 |
| CV (%) | | 40 | 50 | | 91 | 64 | 95 | 95 | 46 | 46 | 38 |

C2, second course; $C_{max}$, maximum concentration; AUC; area under the curve; $T_{max}$, time to maximum concentration. Total = lactone plus acid concentrations. Fed, after breakfast.

bioavailability from lactone is 11.3 ± 5.2% compared with an absolute bioavailability of 11.8 ± 4.5% for total GI147211 (Table 3). The ratio of lactone to total GI147211 after intravenous dosing was similar to the ratio after oral administration. The median intrapatient variability of the absolute bioavailability was 31% (range 3–88%).

At the highest dose level of 6.0 mg m$^{-2}$, the influence of fasted or fed state in absorption of the drug was studied in eight patients. The AUC after fasting was 15.3 ± 8.1 ng h ml$^{-1}$ and, after a breakfast, 16.3 ± 9.7 ng h ml$^{-1}$ ($P = 0.36$, NS).

After oral administration, the terminal half-life of GI147211 lactone ranged from 2.0 to 13.0 h (mean 6.8 ± 3.1 h) and were of the same magnitude as after intravenous administration (mean 8.1 ± 4.1 h) ($P = 0.04$).

### Responses

Tumour responses were evaluable in 17 patients. In two patients, tumour response could not be analysed because of early withdrawal. Best response to treatment was stable disease in seven patients. Short-lasting stable disease occurred in five patients with colon cancer, in one patient with adenocarcinoma of unknown primary and one patient with sarcoma.

### DISCUSSION

The characterization of the inhibition of topoisomerase I as the mechanism of action of CPT has resulted in the development of several semisynthetic CPT analogues, of which some are under extensive clinical investigation. This is the first clinical bioavailability study of orally administered GI147211.

In preclinical studies, absolute bioavailability of GI14721 was 2–5% in mice and 16% in dogs. In the present study, in humans the absolute bioavailability averaged 11.3 ± 5.2%. In comparison, bioavailability studies of topotecan showed a variable systemic exposure of 32% and 44%, which is higher than the bioavailability of oral GI147211 (Kuhn et al, 1995; Schellens et al, 1996). The bioavailability after oral administration of GI147211 showed wide interpatient variability ranging from 4.8% to 24.3%. Intrapatient variability however was more limited.

There was little difference in the ratio of lactone to total GI147211 between oral and intravenous dosing, indicating that the

**Table 4** Pharmacokinetic parameters of GI147211 lactone in 13 patients receiving 6.0 mg m$^{-2}$ oral solution doses in fasted state and fed state. Eight patients were analysed in fasted and fed states.

| Patient | i.v. Dose (mg m$^{-2}$) | i.v. AUC (ng h ml$^{-1}$) | i.v. $t_{1/2}$ (h) | Oral dose | | Oral $T_{max}$ | | Oral AUC | | Oral $t_{1/2}$ | | Absolute bioavailability | |
|---|---|---|---|---|---|---|---|---|---|---|---|---|---|
| | | | | Fasted | Fed | Fasted | Fed | Fasted | Fed | Fasted | Fed | Fasted (%) | Fed (%) |
| 5 | 1.2 | 68.73 | 5.6 | 6 | – | 2 | – | 83.40 | – | 4.2 | – | 24.3 | – |
| 7 | 1.2 | 33.80 | 7.0 | 6 | – | 0.75 | – | 16.56 | – | 4.5 | – | 9.8 | – |
| 9 | 1.2 | 32.71 | 9.5 | 6 | Fed | 0.5 | 0.5 | 24.00 | 31.78 | 4.3 | 8.7 | 14.7 | 19.4 |
| 10 | 1.2 | 40.11 | 10.9 | 6 | – | 1 | – | 10.80 | – | 6.1 | – | 5.4 | – |
| 11 | 1.2 | 34.40 | 10.5 | 6 | Fed | 0.25 | 0.75 | 29.32 | 24.80 | 10.4 | 6.0 | 17.0 | 14.4 |
| 12 | 1.2 | 24.41 | 8.9 | 6 | Fed | 0.25 | 0.75 | 10.39 | 24.80 | 9.7 | 6.0 | 8.5 | 14.4 |
| 13 | 1.2 | 30.29 | 6.8 | 6 | – | 0.5 | – | 8.12 | – | 7.8 | – | 5.4 | – |
| 14 | 1.2 | 27.12 | 4.5 | 6 | Fed | 0.5 | 0.75 | 15.07 | 14.26 | 6.2 | 11.1 | 11.1 | 10.5 |
| 15 | 1.2 | 40.73 | 18.6 | 6 | Fed | 0.5 | 1 | 18.82 | 9.68 | 13.0 | 11.9 | 9.2 | 4.8 |
| 16 | 1.2 | 30.03 | 14.6 | 6 | – | 0.5 | – | 22.88 | – | 6.7 | – | 15.2 | – |
| 17 | 1.2 | 20.75 | 7.2 | 6 | Fed | 0.25 | 1 | 10.70 | 12.37 | 4.3 | 4.1 | 10.3 | 11.9 |
| 18 | 1.2 | 15.03 | 1.9 | 6 | Fed | 0.5 | 0.25 | 6.78 | 3.62 | 5.0 | 3.4 | 9.0 | 4.8 |
| 19 | 1.2 | 19.11 | 4.9 | 6 | Fed | 1 | 0.5 | 7.53 | 9.07 | 3.7 | 3.5 | 7.9 | 9.5 |
| Mean | | 32.09 | 8.53 | | | 0.65 | 0.69 | 20.34 | 16.30 | 6.61 | 6.84 | 11.4 | 11.2 |
| s.d. | | 13.45 | 4.45 | | | 0.47 | 0.26 | 20.21 | 9.71 | 2.87 | 3.36 | 5.2 | 5.0 |
| CV (%) | | 42 | 52 | | | 72 | 38 | 99 | 60 | 43 | 49 | 46 | 44 |

Fasted, fasted state; fed, after breakfast; AUC, area under the curve; $T_{max}$, time to maximum concentration.

acid metabolite is not formated during the first pass. $T_{max}$ of oral GI147211 was 0.5 h or less in 13 of 19 cases, indicating rapid absorption and this was not influenced by the presence of food. Oral dosing of GI147211 appeared to have similar blood half-lives to the intravenous formulation, indicating no prolonged absorption of the oral drug. In conclusion, the absolute bioavailability after administration of an oral solution of GI147211 was low and showed wide interpatient variability. Oral GI147211 bioavailability was not dose dependent and was not affected by the presence of food. It was not possible in this study to determine the contributions of first-pass metabolism vs incomplete absorption to GI147211 bioavailability.

At an oral dose of 6.0 mg m$^{-2}$ day$^{-1}$ GI147211 on day 1 followed by injection of the drug at the dose of 1.2 mg m$^{-2}$ day$^{-1}$ on days 2–5, the onset of neutropenia CTC grade III–IV occurred between day 7 and 19, with a nadir count ranging from 0.09 to 0.98 × 10$^{9}$ l$^{-1}$. The day of the platelet nadir was 8 days, ranging from day 3 to day 16, and the value of CTC grade III–IV thrombocytopenia ranged from 4 to 40 × 10$^{9}$ l$^{-1}$. In contrast to the findings in our phase I study on intravenous GI147211, the current study shows CTC grade III–IV myelotoxicity occurring in patients who have been heavily pretreated (Gerrits et al, 1996).

In other patients, mild leucopenia (CTC grade I–II) with slow recovery frequently occurred, and subsequent courses had to be postponed for 1 week in 10 out of 30 courses at the dose level of 6.0 mg m$^{-2}$, irrespective of pretreatment of patients. Treatment courses with CTC grade III–IV myelosuppression were all uneventful except for one patient with septicaemia.

In topotecan studies, the dose-limiting toxicity was also non-cumulative myelosuppression, predominantly a severe neutropenia of brief duration not necessitating treatment delays (Rowinsky et al, 1992; Verweij et al, 1993). Thrombocytopenia and anaemia occurred mainly in regimens with prolonged intravenous topotecan administration (Hochster et al, 1994; Creemers et al, 1996).

A single oral administration of GI147211 did not result in diarrhoea. No human data are available on effects on the intestinal mucosa with repeated oral GI147211.

Unlike GI147211, which is the active compound, CPT-11 is a prodrug. CPT-11 has to be converted to the active metabolite SN-38. It has been hypothesized that biliary excretion of SN-38 induces diarrhoea as a result of a secretory and exudative mechanism. With oral 9-nitro-camptothecin administration, 33% of patients developed CTC grade ≥ II diarrhoea (Verschraegen et al, 1996).

Preclinical data have indicated that topoisomerase I inhibitors, like topoisomerase II inhibitors, demonstrate more efficacy with prolonged continuous exposure (Houghton et al, 1995).

An oral administration would be most convenient for prolonged dosing. Because of the low absolute bioavailability of GI147211 and the wide range in the interpatient variation, resulting in a non-predictable level of individual drug exposure, development of an oral formulation seems unattractive. The intravenous route is advised for further development of this active and promising new topoisomerase I inhibitor.

### REFERENCES

Aller P, Rius C, Mata F, Zorilla A, Carbanas C, Bellon T and Bernabeu C (1992) Camptothecin induces differentiation and stimulates the expression of differentiation-related genes in U-937 human promonocytic leukemia cells. *Cancer Res* **52**: 1245–1251

Andoh T, Ishii K, Suzuki Y, Ikegami Y, Kusunoki Y, Takemoto Y and Okada K (1987) Characterization of a mammalian mutant with a camptothecin-resistant DNA topoisomerase I. *Proc Natl Acad Sci USA* **84**: 5565–5569

Bendixen C, Thomsen B, Alsner J and Westergaard O (1990) Camptothecin-stabilized topoisomerase I–DNA adducts cause premature termination of transcription. *Biochemistry* **12**: 5613–5619

Camilloni G, Di Martino E and Di Mauro E (1989) Regulation of the function of eukaryotic DNA topoisomerase I: topological conditions for inactivity. *Proc Natl Acad Sci USA* **86**: 308–3084

Champoux J (1976) Evidence from an intermediate with a single strand break in the reaction catalyzed by the DNA untwisting enzyme. *Proc Natl Acad Sci USA* **73**: 3488–3491

Creaven PJ, Allen LM and Muggia FM (1972) Plasma camptothecin (NSC 100880) levels during a 5-day course of treatment: relation to dose and toxicity. *Cancer Chem Rep* **56**: 573–578

Creemers GJ, Lund B and Verweij J (1994) Topoisomerase I inhibitors: topotecan and irinotecan. *Cancer Treat Rev* **20**: 73–96

© Cancer Research Campaign 1997

Creemers GJ, Gerrits CJH, Schellens JHM, Planting AST, Van Der Burg MEL, Van Beurden V, De Boer-Dennert M, Harteveld M, Loos W, Hudson I, Stoter G and Verweij JJ (1996) Phase II and pharmacologic study on topotecan administered as a 21-days continuous infusion to patients with colorectal cancer. *J Clin Oncl* **9**: 2540–2545

Emerson DL, Vuong A, McIntyre MS, Croom D and Besterman JM (1993) In vivo efficacy of two new water-soluble camptothecin analogs in the human cancer xenograft model. *Proc Am Assoc Cancer Res* **34**: 419

Emerson DL, Besterman JM, Braun R, Evans MG, Leitner PP, Luzzio MJ, Shaffer JE, Sternbach DD, Uehling D and Vuong A (1995) In vivo antitumor activity of two new seven-substituted water-soluble camptothecin analogues. *Cancer Res* **55**: 603–609

Eng WK, McCabe FL, Tan KB, Mattorn MR, Hofmann GA, Woessner RP, Hertzberg RP and Johnson RK (1990) Development of a stable camptothecin-resistant subline of P388 leukemia with reduced topoisomerase I content. *Mol Pharmacol* **38**: 471–480

Gerrits CJH, Creemers GJ, Schellens JHM, Wissel PS, Planting AST, Kunka R, Selinger K, De Boer-Dennert M, Marijnen Y, Harteveld M and Verweij J (1996) Phase I and pharmacologic study of the new topoisomerase I inhibitor GI147211, using a daily ×5 intravenous administration. *Br J Cancer* **73**: 744–750

Giovanella BC, Stehlin JS, Wall ME, Wani MC, Nicholas AW, Liu LF, Silber R and Potmesil M (1989) DNA topoisomerase I-targeted chemotherapy of human colon cancer in xenografts. *Science* **246**: 1046–1048

Gottlieb JA, Guarino AM, Call JB, Oliverio VT and Block JB (1970) Preliminary pharmacologic and clinical evaluation of camptothecin sodium (NSC 100880). *Cancer Chem Rep* **54**: 461–470

Gupta RS, Gupta R, Eng B, Lock RB, Ross WE, Hertzberg RP, Caramfam J and Johnson RK (1988) Camptothecin-resistant mutants of Chinese hamster ovarian cells containing a resistant form of topoisomerase I. *Cancer Res* **48**: 6404–6410

Hirabayashi N, Kim R, Nishiyama M, Aogi K, Saeki S, Toge T and Okada K (1992) Tissue expression of topoisomerase I and II in digestive tract cancers and adjacent normal tissues. *Proc Am Assoc Cancer Res* **33**: 436

Hochster H, Liebes L, Speyer J, Gorich J, Taubes B, Oratz R, Wernz J, Chachoua A, Raphael B, Vinci RZ and Blum RH (1994) Phase I trial of low-dose continuous topotecan infusion in patients with cancer: an active and well-tolerated regimen. *J Clin Oncol* **12**: 553–559

Houghton PJ, Chesine PJ, Hallman JD, Lutz L, Friedman HS, Danks MK and Houghton JA (1995) Efficacy of topoisomerase I inhibitors, topotecan and irinotecan administered at low dose levels in protracted schedules to mice bearing xenografts of human tumors. *Cancer Chemother Pharmacol* **36**: 393–403

Hsiang YH and Liu LF (1988) Identification of mammalian DNA topoisomerase I as an intra cellular target of the anticancer drug camptothecin. *Cancer Res* **48**: 1722–1726

Hsiang YH, Hertzberg R, Hecht S and Liu LF (1985) Camptothecin induces protein-linked DNA breaks via mammalian DNA topoisomerase I. *J Biol Chem* **260**: 14873–14878

Kaufmann WK, Boyer JC, Estabrooks LL and Wilson SJ (1991) Inhibition of replicon initiation in human cells following stabilization of topoisomerase–DNA cleavable complexes. *Mol Cell Biol* **11**: 3711–3718

Kharbanda S, Rubin E, Gunji, Hinz H, Giovanella B, Pantazis P and Dufe D (1991) Camptothecin and its derivatives induce expression of the c-jun-protooncogene in human myeloid leukemia cells. *Cancer Res* **51**: 6636–6642

Kuhn J, Rizzo J, Eckhardt J, Fields S, Cobb P, Rodriguez G, Rinaldi D, Drengler R, Smith L, Peacock, Thurman A, De La Cruz P, Hodges S, Von Hoff D and Burris H (1995) Phase I bioavailability study of oral topotecan (abstract 1538). *Proc Am Assoc Clin Oncol* **14**: 474

Liu LF (1989) DNA topoisomerase poisons as antitumor drugs. *Annu Rev Biochem* **58**: 351–375

Muggia FM, Creaven PJ, Hansen HH, Cohen MH and Selanrig OS (1972) Phase I clinical trial of weekly and daily treatment with camptothecin (NSC 100880): correlation with preclinical studies. *Cancer Chem Rep* **56**: 515–521

Muller M (1985) Quantitation of eukaryotic topoisomerase I reactivity with DNA. Preferential cleavage of supercoiled DNA. *Biochim Biophys Acta* **824**: 263–267

Muller M, Pfund W and Mehta V (1985) Eukaryotic type I topoisomerase is enriched in the nucleolus and catalytically active on ribosomal DNA. *EMBO J* **4**: 1237–1243

Nakaya K, Chou S, Kaneko M and Nakamura Y (1991) Topoisomerase inhibitors have potent differentiation-inducing activity for human and mouse myeloid leukemia cells. *Jpn J Cancer Res* **82**: 184–191

National Cancer Institute (1988) *Guidelines for Reporting of Adverse Drug Reactions*. Division of Cancer Treatment, National Cancer Institute: Bethesda, MD, USA

Potmesil M (1994) Camptothecins: from bench research to hospital wards. *Cancer Res* **54**: 1431–1439

Potmesil M, Hsiang YH, Liu LF, Bank B, Grossberg M, Kirschenbaum S, Forlenza TJ, Penziner A, Kanganis D, Knowles D, Traganos F, and Silber R (1988) Resistance of human leukemic and normal lymphocytes to drug-induced DNA cleavage and low levels of DNA topoisomerase I. *Cancer Res* **48**: 35380–3543

Rowinsky EK, Grochow LB, Hendricks CB, Ettinger DS, Forastiere AA, Horowitz LA, McGuire WP, Sartorius SE, Lubejko BG, Kauf-Mann SH and Donehower RC (1992) Phase I and pharmacologic study of topotecan: a novel topoisomerase I inhibitor. *J Clin Oncol* **10**: 647–656

Schellens JHM, Creemers GJ, Beijnen JH, Rosing H, McDonald M, Davies B and Verweij J (1996) Bioavailability and pharmacokinetics of oral topotecan, a new topoisomerase I inhibitor. *Br J Cancer* **73**: 1268–1271

Slichenmyer WJ, Rowinsky EK, Donehower RC and Kaufmann SH (1993) The current status of camptothecin analogues as antitumor agents. *J Natl Cancer Inst* **85**: 271–291

Stafford CG and St Claire III RL (1995) High-performance liquid chromatographic analysis of the lactone and carboxylate forms of a topoisomerase I inhibitor (the antitumor drug GI147211) in plasma. *J Chromatogr* **663**: 119–126

Sugimoto Y, Tsukahara S, Oh-Hara T, Isoe T and Tsurno T (1990) Decreased expression of topoisomerase I in camptothecin-resistant tumor cell lines as determined by monoclonal antibody. *Cancer Res* **50**: 6925–6930

Tanizawa A and Pommier Y (1992) Topoisomerase I alteration in a camptothecin-resistant cell line derived from Chinese hamster DC3F cells in culture. *Cancer Res* **52**: 1848–1854

Verschraegen CF, Natelson E, Giovanella B, Kavanagh JJ, Freed Man RS, Kudalka AP, Edwards CL and Stehlin J (1986) Phase I study of oral 9-Nitrocamptothecin (9NC). *Proc Am Soc Clin Oncol* **15**: 482

Verweij J, Lund B, Beijnen J, Planting A, De Boer-Dennert M, Koier I, Rosing H and Hansen H (1993) Phase I and pharmacokinetics study of topotecan, a new topoisomerase I inhibitor. *Ann Oncol* **4**: 673–678

World Health Organization (1979) *WHO Handbook for Reporting Results of Cancer Treatment* (1979). WHO Offset Publication no. 40: Geneva, Switzerland

Wyllie AH and Duvall E (1992) Cell injury and death. In *Oxford Textbook of Pathology: Principles of Pathology* Vol 1, McGee O'D, Isaacson PG and Wright NA. (eds), pp. 141–157, Oxford University Press: New York

© Cancer Research Campaign 1997